# Administration of Ether to Infants

**Published:** 1897-06

**Authors:** 


### Administration of Ether to Infants.

Infants are readily brought under its influence without the slightest cyanosis. The young administrator may be puzzled at first how to estimate the degree of anesthesia produced in so young a subject. The writer has found the following method of great service: Place the index finger in the infant's hand, and it will be found that during the earlier part of the administration the finger is grasped very tightly, the palmar reflex being active; but as insensibility approaches, the infant's fingers gradually relax, and so soon as they become lissom the operation may be commenced. As in the case of adult patients, our guide is the conjunctival reflex, so in the infant one should devote his constant attention to the amount of palmar and digital reflex action present. This method is preferable to the conjunctival test, because the conjunctiva of a baby loses its sensitiveness very quickly after being touched a few times with the finger. The infant may appear to be thoroughly insesible by the conjunctival test when it is actually on the point of recovering from the anesthetic, and under such conditions the reapplication of the face-piece will cause struggling, to the great inconvenience of the operator. The writer has never seen bronchitis due to the administration of ether produced in a baby, but he is exceedingly careful to guard against cold air being breathed for several hours after the operation. The opening of a window is strictly forbidden, and when possible the baby is not even removed from the room in which the operation has been performed for at least six hours, and he finds that when these directions are strictly followed there is no danger of bronchitis occurring; moreover, the tendency (if any) to sickness is greatly diminished, and this he attributes to the non-stimulation by cold air of the nerves supplying the lungs with the consequent reflex action on the stomach.—*Pediatrics.*

The production of aluminum in the United States has increased from 168,000 pounds in 1891 to 1,300,000 pounds in 1896, the price in the same time declining from $8 to forty cents a pound.

